# Water-Based Epidemiological Investigation of Hepatitis E Virus in South Africa

**DOI:** 10.1007/s12560-024-09596-1

**Published:** 2024-04-13

**Authors:** Karabo Salemane, Leanne Z. Coetzee, Gina Pocock, Bettina Genthe, Maureen B. Taylor, Janet Mans

**Affiliations:** 1 Department of Medical Virology, School of Medicine, Faculty of Health Sciences, University of Pretoria, Private Bag X323, Gezina, Pretoria, 0031 South Africa; 2 Waterlab, Techno Park, 23B De Havilland Cres, Persequor, Pretoria, 0020 South Africa; 3 Stellenbosch University, Private Bag X1, Matieland, Stellenbosch, 7602 South Africa

**Keywords:** Genotyping, HEV, Piggery, Surface Water, Wastewater

## Abstract

Hepatitis E virus (HEV) is an emerging zoonotic pathogen that exhibits great host diversity. The primary means of transmission of the virus in low- and middle-income countries is contaminated water, often due to a lack of access to proper sanitation, which leads to faecal contamination of water sources. Environmental surveillance is an important tool that can be used to monitor virus circulation and as an early warning system for outbreaks. This study was conducted to determine the prevalence and genetic diversity of HEV in wastewater, surface water (rivers and standpipe/ablution water), and effluent from a piggery in South Africa. A total of 536 water samples were screened for HEV using real-time reverse transcription-polymerase chain reaction. Overall, 21.8% (117/536) of the wastewater, river, and ablution water samples tested positive for HEV, whereas 74.4% (29/39) of the samples from the piggery tested positive. Genotyping revealed sequences belonging to HEV genotypes 3 (98%, 53/54) and 4 (2%, 1/54), with subtypes 3c, 3f, and 4b being identified.

## Introduction

Hepatitis E virus (HEV) is a pathogen of global importance as it is the major cause of acute viral hepatitis (Hoofnagle et al., 2012; Sridhar et al., 2017), surpassing hepatitis A virus (HAV) (Rein et al., 2012; Wong et al., 2021). The World Health Organization (WHO) estimates that HEV causes approximately 20 million infections, more than 44,000 deaths, and 3,000 stillbirths each year (Baez et al., 2017; Rein et al., 2012; Wong et al., 2021). Hepatitis E virus infection is typically self-limiting and presents with comparable clinical manifestations to HAV, such as fever, discomfort, body aches, vomiting, jaundice, and nausea. However, its primary distinguishing characteristic is its increased morbidity and case fatality rate in young adults and pregnant women (Kmush et al., 2013).

Hepatitis E virus belongs to the *Hepeviridae* family and is classified within the genus *Paslahepevirus*, the species *Paslahepevirus balayani* (Purdy et al., 2022). Eight HEV genotypes (HEV-1 – HEV-8) have been described to date (Smith et al., 2020). Genotypes 1 and 2 are exclusively associated with human infections through faecal-oral transmission. Genotypes 3 and 4 are zoonotic in nature and can cause chronic infection in the immunocompromised (Aslan & Balaban, 2020). The predominant reservoirs for HEV-3 include swine, rabbits, deer, and mongoose while the main reservoirs for HEV-4 are humans and swine (Sridhar et al., 2017). Genotypes 5 and 6 have been identified only in animals, specifically wild boars. Genotypes 7 and 8 are novel genotypes which have been recently isolated from camelids (Woo et al., 2014, 2016). Genotype 7, which was identified in dromedaries, could have a significant impact on human health as it was detected in a liver transplant patient who developed chronic HEV infection after consuming camel meat and camel-derived products (Lee et al., 2016). To date, scientists have identified more than 30 HEV subtypes (Smith et al., 2020). Only a few of these subtypes, such as subtype HEV-3c, 3f, 4b, and 4c, are of major clinical importance as they have been identified as key drivers of zoonotic transmission or severe clinical manifestations such as acute liver failure (Abravanel et al., 2020; Hakze-van der Honing et al., 2011; Nakano et al., 2018; Sato et al., 2020).

Hepatitis E virus can be transmitted through two main routes: the faecal-oral route, which involves the consumption of faecally contaminated water, and zoonotic transmission, which occurs when humans consume raw or undercooked meat from HEV-infected animals such as swine, deer and wild boar (van der Poel & Rzezutka, 2017). Person-to-person transmission is relatively infrequent but there have been reported cases of mother-to-infant transmission and transmission through solid organ transplants and blood transfusions (Arankalle & Chobe, 2000; Kumar & Sarin, 2013; Kumar et al., 2001; Murkey et al., 2017).

The presence of HEV in water is a growing concern as both drinking and irrigation water can be contaminated, causing a further spread of HEV through fresh produce and potable water consumption (Kokkinos et al., 2017; Salvador et al., 2020; Tripathy et al., 2019). Modes of water contamination may differ depending on the region but typical contamination scenarios include conditions such as heavy rains, water flowing through contaminated soil or leaking sewage pipes, and living in population-dense areas with no access to safe water supply (Khuroo, 2011). Studies in America, Asia and Europe have demonstrated the presence of HEV in groundwater, rivers, and wastewater (Baez et al., 2017; Takuissu et al., 2022; van der Poel & Rzezutka, 2017). Molecular analysis of wastewater runoff and faeces from pig farms in North Carolina, United States of America (USA), presented evidence of HEV RNA in these samples. Waste from pig farms is often disposed of through land application such as fertilisation and irrigation of crops, which means it could inadvertently seep into groundwater that is used for drinking, resulting in HEV exposure of nearby communities and other pigs on the farms (Kase et al., 2009; Meester et al., 2021). Employment at pig farms and wastewater treatment works (WWTWs) may also be considered a risk factor for HEV transmission (Beyer et al., 2020).

Presently, there is a paucity of data regarding the prevalence and diversity of HEV in South Africa (SA). Early studies in SA indicated a modest seroprevalence of 2.4% in 1994 and then 5.8–14.3% by 1996, suggesting persistent virus circulation and endemicity (Grabow et al., 1994, 1996). More recent studies have revealed a significant increase in the seroprevalence of HEV among blood donors as well as an increase in the incidence and genetic diversity of HEV in pigs and pig-derived products (Adelabu et al., 2017; Chauhan & Gordon, 2022; Lopes et al., 2017; Maponga et al., 2020). A linear relationship has also been reported between seroprevalence, age, and geographical area (Tucker et al., 1996). Despite progress in surveillance, characterisation, and risk factor identification of HEV, limited attention has been paid to asymptomatic individuals or those with mild symptoms who may still be shedding the virus but do not seek medical care. A comprehensive understanding of the prevalence and distribution of HEV in a region cannot be obtained based on clinical case data alone. Therefore, this exploratory study investigated the prevalence and diversity of HEV in SA in various water matrices. Water samples from WWTWs, rivers, runoff from selected ablution facilities, and a piggery were screened using real-time reverse transcription-polymerase chain reaction (RT-PCR), and HEV strains were further characterised by genotyping using Sanger sequencing.

## Materials and Methods

### Sample Collection

Systematic water sampling was conducted from February to September 2021; samples (1 L) were collected from selected rivers, influent from WWTWs, and surface runoff at communal standpipe/ablution sites in seven provinces of SA, namely: Free State (FS), Gauteng (GP), Kwa-Zulu Natal (KZN), Limpopo (L), Mpumalanga (MP), North West (NW), and Western Cape (WC). The sampling frequency varied from weekly to biweekly for WWTWs and surface water (river and standpipe/ablution) sites, respectively. Grab sampling was used for all sites except one WWTW site, which used composite sampling. From May to September 2022, 2-L samples of raw wastewater were collected from pig pens, effluent processed through the solid–liquid separation and autothermal thermophilic aerobic digestion (ATAD) system, and water from the adjacent river at a piggery in Tshwane. All the samples were transported in cooler boxes and refrigerated at 4 °C until further processing.

### Virus Recovery

The skimmed milk flocculation protocol as outlined by Falman et al. (2019) was used to recover viruses from the water samples. Briefly, 5% weight/volume (w/v) pre-flocculated skimmed milk solution (2 mL) (Oxoid, Basingstoke, Hampshire, UK) was added to a 200 mL sample, followed by pH adjustment to 3.0–4.0 using 1 M hydrochloric acid (HCl) (Merck KGaA, Darmstadt, Germany) and gentle shaking at 200 revolutions per minute (rpm) at room temperature (20–25 °C) for 2 hours (h). The samples were then clarified by centrifugation (Sorvall® Super T20, du Pont, Wilmington, DE, USA) at 4500 × g for 30 min (min) at 4 °C. The supernatant was decanted, and the pellet was resuspended in 2 mL phosphate-buffered saline (PBS; pH 7.4; Sigma-Aldrich, St. Louis, MO, USA). Aliquots (1 mL) of each recovered virus concentrate were stored at − 20 °C for analysis, while the remaining volumes were stored at − 70 °C.

### Nucleic Acid Extraction

The virus concentrates were subjected to chloroform treatment before extraction to reduce potential PCR inhibitors. Briefly, chloroform (Merck KGaA) (250 μL) was added to 1 mL of the virus concentrate. The solution was vortexed three times for 15 s (sec) and incubated at room temperature for 5 min, followed by centrifugation at 5000× g for 3 min. Following phase separation, the aqueous phase (1 mL) was transferred to a new 2 mL tube. This process was performed once for wastewater and surface water samples and twice for pig farm samples.

Total nucleic acids were extracted from the wastewater and surface water samples using a QIAamp® UltraSens® Virus Kit (QIAGEN, Hilden, Germany) following the manufacturer’s instructions. Nucleic acids were eluted in AVE buffer (100 μL) and stored at − 80 °C. Nucleic acids from recovered piggery and adjacent river samples were extracted using the EMAG® Nucleic Acid Extraction System (bioMérieux, Marcy-l’Étoile, France) following the manufacturer’s instructions, eluted in 100 μL of elution buffer, and then stored at − 80 °C.

### Positive Control Preparation

An HEV-positive stool specimen, kindly provided by Prof. Wolfgang Preiser from Stellenbosch University, SA, was used to generate a positive control plasmid for the detection assay. Total nucleic acids were extracted from a 10% suspension of the stool specimen using the QIAamp® UltraSens® Virus Kit (QIAGEN) according to the manufacturer’s instructions. To construct the plasmid, complementary DNA (cDNA) was synthesised using a Protoscript® II Reverse Transcriptase Kit (New England Biolabs Inc., Ipswich, MA, USA), 0.5 mM deoxynucleotide triphosphates (dNTPs) (New England Biolabs Inc.), and random hexamer primers (Roche, Basel, Switzerland) according to the manufacturer’s instructions. A PCR was then performed using the cDNA (5 μL), EmeraldAmp MAX HS PCR Master Mix (25 μL) (Takara Bio Inc., Kusatsu, Shiga, Japan), nuclease-free water (18 μL; Promega Corporation, Madison, WI, USA), and published primers: JVHEVF and JVHEVR, at a final concentration of 0.4 μM for each primer (Table 1). The thermal cycling parameters were 95 °C for 3 min, followed by 40 cycles at 94 °C for 1 min, 45 °C for 1 min, and 72 °C for 1 min, with a final extension at 72 °C for 7 min.

**Table 1:** Primers and probes for detection and genotyping of HEV

		Primer	Sequence^d^	Type	Location^e^
Detection		JVHEVF^a^	5′-GGTGGTTTCTGGGGTGAC-3′	Forward	5261–5278
		JVHEVR^a^	5′-AGGGGTTGGTTGGATGAA-3′	Reverse	5313–5330
		JVHEVP^b^	5′-FAM-TGATTCTCAGCCCTTCGC-MGB-3′	Probe	5284–5301
Genotyping	1st PCR	MengFO^c^	5′-AAYTATGCMCAGTACCGGGTTG-3′	Forward	5687–5708
		MengRO^c^	5′-CCCTTATCCTGCTGAGCATTCTC-3′	Reverse	6395–6417
	2nd PCR	MengFI^c^	5′-GTYATGYTYTGCATACATGGCT-3′	Forward	5972–5993
		MengRI^c^	5′-AGCCGACGAAATYAATTCTGTC-3′	Reverse	6298–6319

^a^Jothikumar et al*.* (2006)

^b^Modified probe by Garson et al. (2012). Probe labels: 6-carboxy fluorescein (FAM), minor groove binder (MGB)

^c^Huang et al*.* (2002)

^d^Degenerate base code: Y = C or T; M = A or C

^e^The sequence location of all primers and probe corresponds to nucleotide position 5261–6417 of GenBank accession number M73218

The 69 bp product was then purified and cloned into a pJET1.2/blunt vector using the CloneJET™ PCR Cloning Kit (Thermo Fisher Scientific, Waltham, MA, USA) and 10-beta *Escherichia coli* (*E. coli*) chemically competent cells (New England Biolabs Inc.). Following colony PCR, the plasmid was purified using the Zyppy™ Plasmid Miniprep Kit (Zymo Research, Irvine, CA, USA) and stored at − 20 °C until further use.

### Real-Time RT-PCR Assay for HEV Detection

A one-step real-time RT-PCR assay (QuantiFast Pathogen® RT-PCR + IC kit, QIAGEN) was used to amplify the 69 bp region of the highly conserved ORF3. The nucleic acid template (5 μL), nuclease-free water (7.55 μL; Promega Corp.), 5× Reaction Mix (5 μL), and published primers and probe (Table 1) at a final concentration of 0.4 μM and 0.08 μM respectively, were added to a final reaction volume of 25 μL. The kit’s internal control (IC) was used to monitor PCR inhibition.

The assay was performed using the QuantStudio™ 5 platform (Applied Biosystems, Waltham, MA, USA). The cycling conditions were as follows: reverse transcription at 50 °C for 30 min, enzyme activation at 95 °C for 5 min, and 45 cycles at 95 °C for 15 s and 60 °C for 1 min. Fluorescence was measured during the extension step. The data was analysed with QuantStudio™ software (Applied Biosystems).

A standard curve was constructed using triplicate tenfold serial dilutions of the positive control plasmid (r^2^ = 0.995, efficiency = 94.57%, error = 0.063). The limit of detection (LOD) of the assay, defined as the 95% confidence minimum detectable template concentration, and the limit of quantification (LOQ), defined as the lowest concentration of HEV RNA that could be reliably quantified, were determined based on the standard curve. A cycle threshold (Ct) value of 40 was used as the cut-off value for HEV-positive samples. In instances where substantial IC inhibition was observed, that is when no amplification signals were detected from both the target sequence and the IC or the Ct value of the IC was ≥ 40 with no amplification from the target sequence, the RT-PCR assay was repeated with diluted nucleic acid (1:10 dilution). If the IC tested positive after dilution, inhibition was considered as resolved. Hepatitis E virus RNA in selected positive samples (Ct < 33.9) was quantified using the real-time RT-qPCR assay described above.

### Molecular Characterisation

#### Amplification of the Partial Capsid Region

Samples with a Ct value ≤ 35.5 were selected for molecular characterisation using a two-step RT-PCR. The extracted nucleic acid template (5 μL) was used to synthesise cDNA (20 μL) using random hexamer primers (30 μM) (Thermo Fisher Scientific), 0.5 mM dNTPs (New England Biolabs Inc.), and the Protoscript® II Reverse Transcriptase Kit (New England Biolabs Inc.), according to the manufacturer’s instructions. The 348 bp partial capsid region (ORF2) was amplified by nested PCR using a 50 μL reaction containing cDNA (5 μL), EmeraldAmp MAX HS PCR Master Mix (25 μL) (Takara Bio Inc.), nuclease-free water (Promega Corp.), and published primer sets (Table 1) at a final concentration of 0.2 μM. The first-round PCR product (1 μL) was used as the template for the second round. The cycling conditions for both the first and second rounds were as follows: 95 °C for 3 min, followed by 40 cycles of 94 °C for 1 min, 45 °C for 1 min, and 72 °C for 1 min, with a final extension at 72 °C for 7 min.

#### Visualisation of PCR Products and Purification

Amplicons were analysed by 1.5% agarose gel electrophoresis using Agarose LE (Cleaver Scientific, Rugby, Warwickshire, UK) and purified using the DNA Clean and Concentrator® − 25 Kit (Zymo Research) according to the manufacturer’s instructions. Amplicons with low yields were cloned using the CloneJET™ PCR Cloning Kit (Thermo Fisher Scientific) and 10-beta *E. coli* chemically competent cells (New England Biolabs Inc.) as per the manufacturer’s instructions.

#### Sanger Sequencing

The PRISM BigDye® Terminator v3.1 Cycle Sequencing Kit (Thermo Fisher Scientific) was used for cycle sequencing of amplicons of the correct size. The cycling conditions were as follows: initial denaturation (94 °C for 3 min), followed by 25 cycles of denaturation (94 °C for 30 s), annealing (50 °C for 10 s), and extension (60 °C for 4 min). Sequencing reactions were referred to Inqaba Biotec, Pretoria, SA, for purification and analysis.

### Phylogenetic Analysis

The BioEdit Sequence Alignment Editor Software was used for base calling, while Sequencher™ DNA Sequence Analysis Software version 4.9 (Gene Codes Corporation, Ann Arbor, MI, USA) was utilised for sequence assembly and translation (Hall, 1999). To identify the genotypes, the Nucleotide Basic Local Alignment Search Tool (BLAST) was applied to compare them to known sequences in the GenBank database. The HEV Genotyping Tool version 1.0 (https://www.rivm.nl/mpf/typingtool/hev/) was used to assign the subtypes. All sequences were deposited in GenBank under accession numbers OR604636 – OR604689.

Sequence alignment was performed with the Multiple Alignment using Fast Fourier Transform (MAFFT) version 7 software (https://mafft.cbrc.jp/alignment/server/) (Katoh et al., 2019). The derived HEV sequences were aligned to GenBank top hits and HEV reference subtypes as recommended by Smith et al. (2020). A maximum-likelihood phylogenetic tree was constructed by using the Molecular Evolutionary Genetics Analysis Program Version X (MEGA X) software; the evolutionary distances were determined using the Jukes-Cantor method and validated by replication with 1000 bootstraps.

### Statistical Analysis

Descriptive statistics were used to summarise HEV detection rates. The significance of differences in HEV detection rate according to sample type, province, and time period were assessed using the chi square test on OpenEpi (Sullivan et al., 2009) (https://www.openepi.com/TwobyTwo/TwobyTwo.htm, accessed 21/02/2024).

## Results

### Hepatitis E Virus Detection

A total of 536 water samples were collected from February to September 2021. Of these, 328 were influent samples from WWTWs, 188 were river samples, and only 20 were from standpipe/ablution sites. Hepatitis E virus was detected in 21.8% (117/536) of the samples, with detection rates of 22% (72/328) in wastewater influent, 22% (42/188) in river water, and 10% (2/20) in ablution runoff/standpipe site samples. The average Ct value for the positive samples was 34.5 (WWTWs), 34.6 (rivers), and 36.8 (standpipe/ablution). No statistically significant difference was observed in the detection rates in wastewater influent and river water (p = 0.3382). Inhibition was observed in 18.9% (98/536) of samples. After dilution, 14.3% (14/98) tested positive (average Ct value: 36.28) while 72% (71/98) were negative and 13 samples (2.4%) remained inconclusive. In total, 406 samples were negative. The LOD of the real-time RT-qPCR assay was determined as 100 genome copies (gc)/reaction and the LOQ as 1000 gc/reaction. The HEV RNA concentrations for the positive samples ranged between 1.89 × 10^3^ gc/reaction and 2.35 × 10^4^ gc/reaction.

**Fig. 1:**
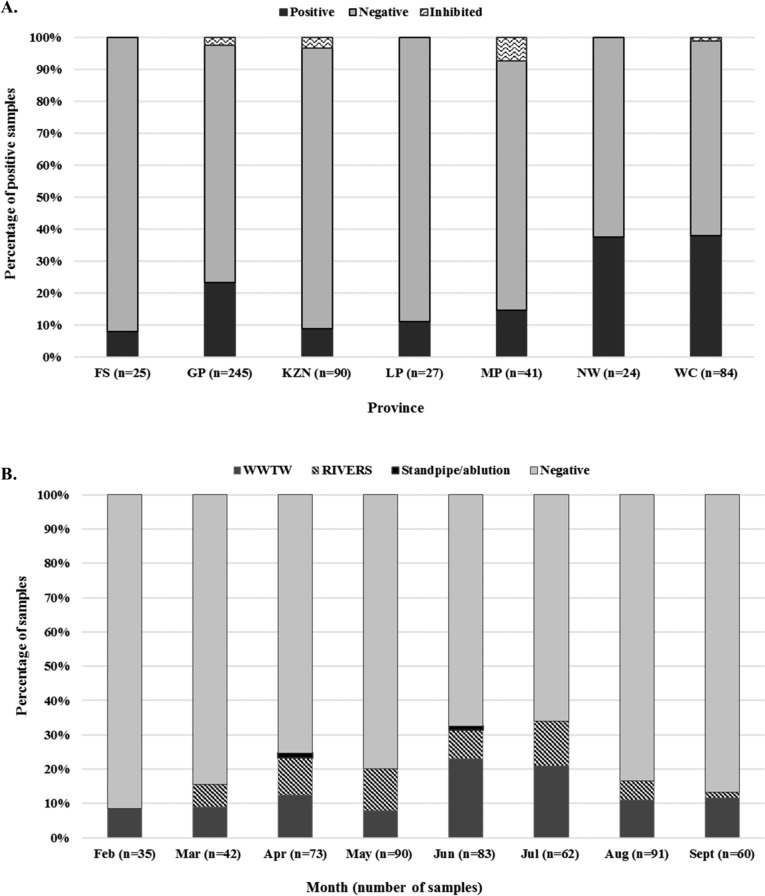
The number of HEV-positive, negative, and inhibited samples collected between February to September 2021 per province (**A**) and the distribution of HEV-positive samples through the sampling period (**B**). Figure created in Microsoft Excel 2016

Hepatitis E virus was detected in all seven provinces with the WC having the highest percentage of positive samples (38.1%, 32/84), followed by NW (37.5%, 9/24) and then GP (23.3%, 57/245) (Fig. 1A). The HEV detection rate in the WC was significantly higher than all other provinces (p < 0.006) except for NW (p = 0.4627). A higher frequency of positive samples was observed in the winter months of June and July, with a progressive decrease in positivity from August (Fig. 1B). No significant difference was observed from the samples in the WC (p > 0.05) between February-April (30% positivity), May–July (45% positivity), and August–September (40% positivity). However, GP demonstrated a statistically significant increase in positive samples (p < 0.02) during May–July (31% positivity) compared to both the February-April (14% positivity) and August–September (15% positivity) time periods.

Between May and September 2022, an additional 39 samples were collected from the Tshwane piggery and the adjacent river. These included 10 samples of raw effluent, 10 samples of effluent after settling, 9 samples of effluent after separation, and 10 samples from the adjacent river. All effluent samples from the piggery (29) tested positive for HEV (average Ct value: 27.5) with RNA concentrations ranging between 1.13 × 10^4^ gc/reaction and 1.79 × 10^6^ gc/reaction, while all samples from the river adjacent to the piggery (10) tested negative.

### Molecular Characterisation

#### Amplification of the Partial Capsid Region

Of the 74 WWTW influent and surface water samples selected for genotyping (average Ct value: 33.18), the HEV partial capsid region was successfully amplified in 33.8% (25/74) of the samples. Additionally, 100% (29/29) of the HEV-positive piggery effluent samples were successfully amplified (average Ct value: 27.54). A total of 54 HEV strains were sequenced, genotyped, and phylogenetically analysed.

#### Phylogenetic Analysis

**Fig. 2:**
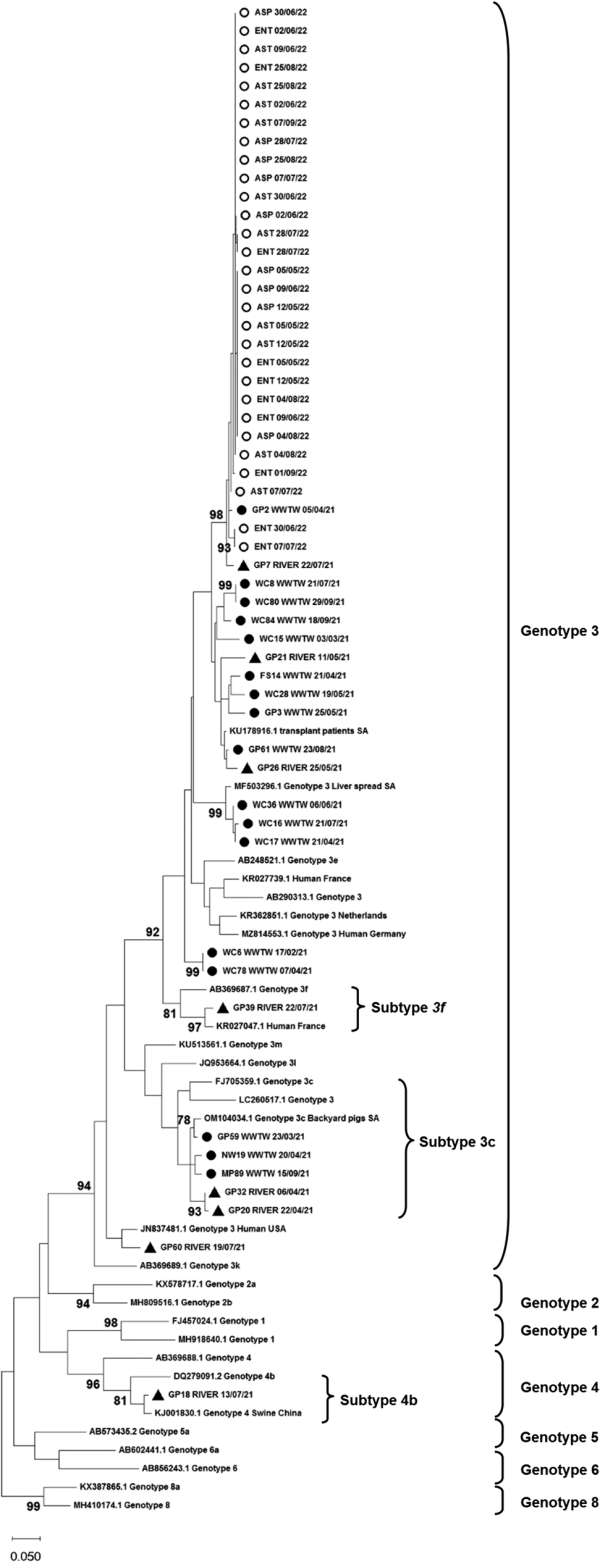
Maximum-likelihood phylogenetic tree of partial capsid gene sequences (348 bp, ORF2) representing the 54 HEV strains detected in the study. Evolutionary distances were determined using the Jukes-Cantor method, conducted in MEGA X. Numbers next to the branches indicate nodes where bootstrap support was > 70% out of the 1000 replicates. Reference sequences are based on Smith et al. (2020). Closely related strains from GenBank are indicated by accession numbers and study strains are indicated by triangles ▲(river), solid circles ● (WWTWs), and open circles ○ (piggery)

A maximum likelihood phylogenetic tree was constructed to determine the relatedness of the sequences obtained in this study to those in GenBank (Fig. 2). Most of the strains clustered with strains from SA and were classified as HEV-3 (53/54), with only one strain being identified as HEV-4 (1/54). Further analysis using the HEV typing tool revealed that three HEV-3 strains belonged to subtypes HEV-3f and HEV-3c, and the HEV-4 strain belonged to subtype HEV-4b. The remaining strains (n = 51) were categorically of genotype HEV-3, but the online HEV typing tool was unable to classify them into a subtype.

## Discussion

Wastewater-based epidemiology (WBE) was applied to investigate the prevalence and diversity of HEV in wastewater and other water matrices across both rural and urban settings in SA. The WBE field is a relatively new and dynamic area of research that can complement conventional surveillance systems and serve as an early warning system for infectious disease outbreaks in specific regions (Sims & Kasprzyk-Hordern, 2020).

Real-time RT-PCR was used to detect HEV. This detection method has been used in previous studies from other countries, including Pakistan (Ahmad et al., 2010), Sweden (Hellmér et al., 2014), Portugal (Salvador et al., 2020), and Argentina (Lo Castro et al., 2023). Because of its widespread availability, dependability, and affordability, this method is regarded as the gold standard for virus detection in water matrices. Other assays, such as the reverse transcription droplet digital PCR (RT-ddPCR) assay, have been developed and have demonstrated improved sensitivity, specificity, and reproducibility compared to real-time RT-PCR (Nicot et al., 2016). However, the costs and need for specialised equipment make such assays impractical for most researchers, especially in low- and middle-income countries (LMICs).

Real-time RT-PCR inhibition could not be resolved for 2.4% (13/536) of the wastewater and surface water samples collected from February-September 2021. Compounds present in samples, such as metal ions, lipids, proteins, and polysaccharides, can inhibit nucleic acid amplification (Schrader et al., 2012). Diluting the RNA can reduce the concentration of such compounds, therefore assisting with the amplification of the target. Inhibition was predominantly detected in the wastewater samples, as well as in a few surface water samples extracted manually using the QIAamp® UltraSens® Virus Kit (QIAGEN). No inhibition was observed in the piggery samples collected between May and September 2022, which could be due to the use of a different extraction method (EMAG® Nucleic Acid Extraction System) or the less complex matrix of piggery wastewater with fewer potential PCR inhibitors. Inhibition leads to a possible underestimation of the positivity rate within a sample pool.

A 21.8% HEV positivity rate was found in wastewater and surface water samples collected between February and September 2021. The detection rate in wastewater influent was high (22%), this is likely because significant concentrations of HEV are shed in stool excreted by infected individuals, making it more detectable in wastewater influent, which is a pooled sample from thousands of people in each area (Hellmér et al., 2014; Iaconelli et al., 2020; Takuissu et al., 2022). A recent meta-analysis of HEV in environmental waters showed that its prevalence is higher in untreated wastewater (15.1%) compared to surface waters (7.4%) (Takuissu et al., 2022). Interestingly, the prevalence of HEV in wastewater is typically lower in most industrialised countries, such as Italy (5.4%) (Iaconelli et al., 2020), Norway (8.0%) (Myrmel et al., 2015), and Portugal (3.3%) (Matos et al., 2018) when compared to LMICs.

For the surface water samples collected between February and September 2021, the detection rate of HEV in the rivers and standpipe/ablution sites was 22% and 10%, respectively. These results are comparable to the detection rate in WWTW influent. This study shows frequent HEV contamination of SA river systems at low levels. According to the Green Drop Watch Report for 2023, 70.1% of wastewater treatment plants in SA are dysfunctional or have exceeded their design capacity (Department of Water and Sanitation, Republic of South Africa, 2023). There are also unsewered informal settlements with rapidly changing population numbers close to rivers leading to inadequate disposal of sewage. Many of these informal settlements have backyard pigs and other free-ranging animals which could shed HEV. The lower detection rates of HEV, or lack thereof, in surface water in industrialised countries can be attributed to access to proper sanitation, hygiene, and adequate disposal and treatment of sewage (Ahmad et al., 2022).

All piggery samples (29) tested positive for HEV. Pigs are one of the main reservoirs of HEV (Ahmed & Nasheri, 2023). Infected pigs can shed the virus in their stool, therefore, exposure to manure and faecally contaminated pig pens is a potential source of infection for both pigs and farm workers (Beyer et al., 2020; Meester et al., 2021). The presence of HEV RNA in the environment is not conclusive evidence of infectious virions, but it does highlight the potential risks. Studies have shown that working at WWTWs and piggeries/slaughterhouses as an HEV risk factor (Bagulo et al., 2020; Pavio et al., 2017; Vaidya et al., 2003). In Antioquia, Columbia, pig farm employees had an HEV seroprevalence of 11.25% (Baez et al., 2017). To mitigate the risk of HEV infection in farm workers, it is imperative to provide them with adequate personal protective equipment and to handle all waste carefully. Vaccination of these cohorts for disease prevention would be optimal. However, the only available vaccine that has demonstrated good safety and high efficacy is solely licensed in China and Pakistan since 2011 and 2020, respectively (Zhong et al., 2023).

None of the samples from the river adjacent to the piggery tested positive for HEV. These results align with a study conducted by Kasorndorkbua et al. (2005) in the USA, which suggested that contamination of the adjacent river may not be evident because the virions in the river being present at a low concentration and thus undetectable by the applied detection methods. Alternatively, the effective implementation of aerobic digestion similar to ATAD described by Wi et al. (2019), coupled with the initial release of the effluent into a wetland, likely ensured sufficient treatment of the waste before it reached the river.

Although the seasonality of HEV is unclear (Fares, 2015), an increased incidence of HEV in the summer months of May–July in Europe suggests a summer seasonality of HEV in that region (Healy et al., 2022). Lu and colleagues (2013) reported that the seasonality of HEV differed according to the geographical area in China (Lu et al., 2013), emphasising the complexity of describing HEV seasonality. In this study, HEV was detected throughout the sampling period at varying frequencies across the months and provinces. Analysis of the data revealed a gradual increase in the incidence of HEV from March, with a peak observed in June and July. As the winter season progressed, there was a gradual decrease in the incidence of HEV. Conclusions on the seasonality of HEV in SA could not be drawn from this study because the sample collection duration was too short, however it is clear the virus circulates in the population for most of the year. This suggests that regular environmental and clinical surveillance is required to predict potential outbreaks, especially in densely populated rural areas with limited access to clean water and proper sanitation.

The South African National Health Act (Act No. 61 of 2003) classifies hepatitis E as a category 2 medical condition, requiring health care professionals to report clinical/laboratory confirmed cases to the Department of Health through written or electronic communication within 7 days. However, between June 2018 and May 2023, only 247 cases were reported in the notifiable medical condition system for the entire country (NICD, 2023). Considering the HEV detection rate in the current study, HEV infections are likely underreported or misdiagnosed in the country. Research has shown that most cases of hepatitis E are misdiagnosed as drug-induced liver injury (DILI), especially in LMICs (El-Mokhtar et al., 2021; Kamar et al., 2014). In SA, over five million people were receiving antiretroviral therapy in 2022 (UNAIDS, 2022). Antiretroviral therapy causes DILI in patients with HIV infection (Pillaye et al., 2020), often leading to healthcare workers misdiagnosing HEV-induced liver inflammation as DILI. This is caused by limited HEV awareness in clinical settings. Environmental surveillance is therefore necessary to improve our understanding of the prevalence of HEV in the country. The low prevalence of the virus in the clinical setting could also be due to its low concentrations, causing sporadic, asymptomatic infections as it spreads (Grabow et al., 1996).

Among the 74 positive samples collected between February and September 2021 and selected for genotyping, only 33.8% (25/74) were positive by nested PCR. This low positivity rate may be due to low concentrations of the virus and inhibitors within the sample. All piggery samples collected between May and September 2022 were successfully genotyped. These samples had a 100-fold higher viral concentration than the 2021 samples, which likely facilitated efficient amplification using nested PCR. Phylogenetic analysis revealed the predominance of HEV-3 in the country (98.1% [53/54]), with a single detection of genotype 4 (1.85% [1/54]). Previous investigations in various countries, including Italy (La Rosa et al., 2015, 2017), Germany (Beyer et al., 2020), and Australia (Miura et al., 2016), have reported the presence of these genotypes in similar environmental sources. Nucleotide BLAST analysis indicated that most sequences from this study (74%) were closely related to an HEV-3 sequence identified in a renal transplant patient (KU178916.1) from the WC province in SA. Interestingly, the phylogenetic tree showed that strains from the piggery formed a distinct cluster that was more closely related to strains detected in a WWTW and river in this study than to their top hit from GenBank (renal transplant patient) (Andersson et al., 2015). The samples in this cluster could not be definitively subtyped due to insufficient bootstrap support, as the bootstrap cut-off was 70%. Future studies should characterise a larger region of the genome to determine whether or not these samples belong to a new, possibly unclassified subtype.

One study strain was identified as subtype 3f and clustered with a human strain from France (Lhomme et al., 2015) with a significant bootstrap value (80%). Infection with this subtype correlates with elevated HEV viral titres, a higher fever risk, and increased hospitalisation rates (Abravanel et al., 2020). Three strains (5.6%) were most closely related to an HEV-3 strain detected in pork liver spread (MF503296.1) from SA (Korsman et al., 2019). The remaining sequences (18.5%) matched those from swine in Nigeria (KJ451631.1), Italy (MK532915.1), SA (OM104034.1), Japan (AB094271.1), and China (KJ001830.1). Overall, the sequences from this study showed nucleotide identities between 93.0% and 97.0% with their top hits in GenBank. Within the SA context, there is limited sequence data available for HEV, with only 21 sequences from SA available in GenBank to date.

The subtype 3c strains, related to HEV-3 detected in backyard pigs in KZN (OM104034.1) (Chauhan & Gordon, 2022), were detected in GP, MP and NW. This subtype is widespread in pigs and wild boars (Fenaux et al., 2018). Although 3c infections are associated with a low viral load (Abravanel et al., 2020), a study spanning four European countries, comparing HEV infections in symptomatic and asymptomatic individuals found that infection with 3c leads to more asymptomatic infections (Smith et al., 2015). This could further perpetuate the spread of HEV. HEV strains found in human populations have been detected in water matrices in both industrialised and LMICs (Li et al., 2017). The association of the HEV strains in this study with strains from clinical cases suggests a wider community spread than previously thought. This poses a health risk, especially to immunocompromised people, who may suffer from chronic infection after exposure to the virus in rivers and other water sources.

Our findings are consistent with those of Beyer et al. (2020), who similarly identified both subtypes 3c and 3f in environmental samples in Germany. Subtypes 3c and 3f are predominant in Europe; however, subtype 3f has also been found in Thailand and Japan (Nakano et al., 2018). Infection with subtype 3f is associated with an increase in hospitalisation and a higher viral load, whereas subtype 3c has mostly been implicated in increased HEV incidence. (Abravanel et al., 2020; Nakano et al., 2018). The single HEV 4 strain was classified as subtype 4b and clustered with a strain from a pig in China and a reference strain, as described by Smith et al. (2020) (81% bootstrap support). Infections with genotype 4 have been noted to cause more severe clinical manifestations in humans (Hakze-van der Honing et al., 2011). The phylogenetic findings in this study are in line with most studies that have investigated the genetic diversity of HEV in water matrices, as HEV-3 has been recognised as the most predominant genotype, with HEV-4 being present in low quantities (Iaconelli et al., 2020; Takuissu et al., 2022). A limitation of our study is that we could not confirm whether the viruses identified in this study were infectious or not. Future studies should incorporate viability PCR in their methods to detect viable viruses that could potentially cause infection.

## Conclusion

From the available information, this is the first study of its kind to be conducted in SA. Our analyses present compelling evidence for the presence of HEV in pig slurry, wastewater, and surface waters within SA. Phylogenetic analysis established a clear link between the strains detected in the environment and those previously detected in human cases, indicating an ongoing circulation of HEV in the population that extends beyond reported clinical cases. This study not only expands our understanding of water-based epidemiology, but also significantly contributes to bridging the existing knowledge gap concerning the prevalence and distribution of HEV in our region. Furthermore, the valuable data obtained from this research can inform and support the development of effective vaccines and implementation of preventive measures to mitigate HEV outbreaks.
